# Secure pulmonary diagnosis using transformer-based approach to X-ray classification with KL divergence optimization

**DOI:** 10.3389/fmed.2025.1716066

**Published:** 2025-12-17

**Authors:** Vatsala Anand, Mohammed Shuaib, Irfanullah Khan, Mehran Ullah, Shadab Alam

**Affiliations:** 1 Department of Computer Science and Engineering, Akal University, Talwandi Sabo, Bathinda, Punjab, India; 2 Department of Computer Science, College of Engineering and Computer Science, Jazan University, Jazan, Saudi Arabia; 3 Department of Information Systems and Operations Management, King Fahd Business School, King Fahd University of Petroleum and Minerals, Dhahran, Saudi Arabia; 4 Interdisciplinary Research Center for Smart Mobility and Logistics, King Fahd University of Petroleum and Minerals, Dhahran, Saudi Arabia; 5 School of Business and Creative Industries, University of the West of Scotland, Paisley, United Kingdom

**Keywords:** pulmonary disease classification, secure medical diagnostics, lung disease classification, deep learning, chest X-ray analysis, medical image augmentation

## Abstract

**Introduction:**

Lung disease classification plays a significant part in the early discovery and determination of respiratory conditions.

**Methods:**

This paper proposes a novel approach for lung disease classification utilizing two advanced deep learning models, MedViT and Swin Transformer, applied to the Lung X-Ray Image Dataset that includes 10,425 X-ray images categorized into three classes: Normal with 3,750 images, Lung Opacity with 3,375 images, and Viral Pneumonia with 3,300 images. A series of data augmentation methods, including geometric and photometric augmentation, are applied to improve model performance and generalization.

**Results:**

The results illustrate that both MedViT and Swin Transformer accomplish promising classification accuracy, with MedViT showing particular strength in medical image-specific feature learning due to its hybrid convolutional and transformer design. The impact of different loss functions is also examined, where Kullback-Leibler Divergence yields the highest accuracy and effectively handles class imbalance. The best-performing MedViT model achieves an accuracy of 98.6% with a loss of 0.09.

**Discussion:**

These findings highlight the potential of transformer-based models, particularly MedViT, for reliable clinical decision support in automated lung disease classification.

## 1 Introduction

Lung diseases, including pneumonia, COVID-19, tuberculosis (TB), and lung cancer, represent a few of the most critical public health challenges around the world. These conditions often present with similar side effects, such as coughing, shortness of breath, and fatigue, making an accurate and timely determination critical for effective treatment. Pneumonia and COVID-19 are especially concerning due to their ability to quickly spread and cause serious respiratory complications. Lung cancer, one of the deadliest cancers universally, is frequently analyzed at advanced stages, making early detection essential for improving survival rates (1, 2). Early intervention can altogether improve patient results, diminish the spread of infectious illnesses, and lower the burden on healthcare systems. For example, in COVID-19, early identification of infected people through imaging can help in segregating patients and avoiding broad transmission. Also, in pneumonia and lung cancer, early detection through diagnostic imaging empowers timely medications such as antibiotics, antivirals, or surgery, which can considerably diminish mortality rates (3). However, diagnosing these illnesses precisely, particularly in their early stages, can be challenging due to overlapping symptoms and the subtle nature of a few radiological signs (4–6).

**FIGURE 1:**
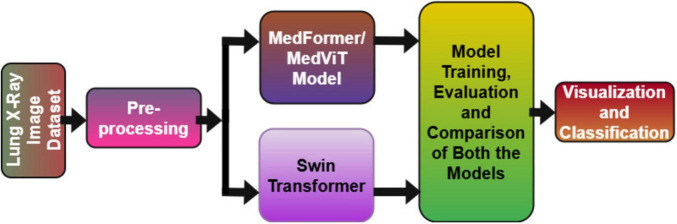
Theme diagram of the study.

As AI frameworks gotten to be more integrated into clinical workflows, ensuring the security and cybersecurity of medical information and diagnostic models has become a basic concern. Deep learning models, especially those that use Convolutional Neural Networks (CNNs) and transformers, have made huge improvements in making medical image analysis more accurate and effective. These models can learn detailed patterns from medical images like chest X-rays, CT scans, and MRI scans, and they can tell the difference between healthy and unhealthy tissues with great precision. By training on huge datasets, deep learning algorithms can help radiologists in recognizing and diagnosing lung diseases like pneumonia, TB, and lung cancer in their early stages, thus decreasing human error and progressing diagnostic workflows. In recent years, transformer-based models like MedViT and Swin Transformer have further progressed the field by leveraging global context and attention mechanisms to progress feature extraction and classification in medical imaging. Motivated by these advancements, this study investigates the application of MedViT and Swin Transformer models for precise classification of lung diseases, aiming to improve early determination and support clinical decision-making through progressed deep learning methods. Figure 1 displays the theme diagram of lung disease classification.

### 1.1 Scientific contributions

1. *Improvement of a hybrid transformer-based model:* Proposed a novel application of MedViT and Swin Transformer structures for lung classification, viably capturing both local and global image features from chest X-rays.
2. *Assessment on multi-class lung disease dataset:* Utilized a comprehensive dataset of 10,425 chest X-ray images characterized into Normal, Lung Opacity, and Viral Pneumonia classes, accomplishing high classification accuracy over all categories.
3. *Advanced loss function investigation:* Conducted a comparative study of different loss functions—including Hinge Loss, Binary Cross-Entropy, and Kullback-Leibler Divergence—demonstrating that KL Divergence gives prevalent performance in dealing with class imbalance.
4. *Improved generalization through data augmentation:* Applied diverse geometric and photometric data augmentation methods to progress model vigor and generalization in real-world clinical scenarios.

### 1.2 Structure of paper

The rest of the paper is arranged as Sect. 2 shows the related work, proposed work in Sect. 3, results in Sect. 4 and conclusion Sect. 5 respectively.

## 2 Related work

In recent years, deep learning has risen as a transformative approach in medical imaging, especially for the automated classification of lung infections utilizing chest X-ray images. A few studies have proposed blockchain-based systems and progressed cybersecurity conventions to upgrade the security and integrity of AI-driven chest X-ray classification systems. Various studies have investigated different convolutional and transformer-based models to improve diagnostic accuracy and clinical decision-making. Hage Chehade et al. (7) presented a CycleGAN model to provide sharper images. Further, they had used the DenseNet121 model with an attention mechanism to focus on relevant areas. They had obtained an AUC of 91.38% on the Chest X-ray dataset. Patel et al. (8) had presented the integration of explainable AI (XAI) for multi-disease classification to achieve an accuracy of 96%. Upasana et al. (9) had presented a modified DenseNet201 model with a hybrid pooling layer to achieve an accuracy value of 95.34% on the chest X-ray dataset. Ashwini et al. (10) had used two classification models for the recognition and classification of lung disease. They obtained an accuracy value of 98.75 while working on different disease classes, namely TB, COVID-19, pneumonia, lung cancer, and lung_opacity. Shamrat et al. (11) had presented a modified MobileNetV2 model for the detection and diagnosis of lung disease from chest X-ray images. They had obtained the value of accuracy as 91.6%. Kuzinkovas et al. (12) had introduced an ensemble pre-trained model for the detection of lung disease with GLCM features. The ensemble model has attained 98.34% accuracy with different number of images. Ravi et al. (13) had used EfficientNetB0, B1, and B2 models and fused the features of the models. They had achieved the value of accuracy between 98 and 99%. Mann et al. (14) presented three pre-trained lung disease detection models with the ChestX-ray dataset, achieving an AUC of 0.9450 for the DenseNet121 model. Huy et al. (15) had presented CBAMWDnet for the detection of TB in chest X-ray images. They had worked on a total of 5,000 images and had achieved a value of accuracy of 98.80%. Putri et al. (16) had used K-means clustering for the classification of lung disease. They had also used the Canny edge detection method for the detection of the thickness of edges. They had worked on 1,991 X-ray images and had achieved an accuracy value of 73%. Singh et al. (17) presented a Quaternion Channel-Spatial Attention Network for the classification of ChestX-Ray images. They had worked using 5,856 ChestX-Ray images and had achieved an accuracy value of 94.53%. Tekerek et al. (18) had used MobileNet and DenseNet models for chest X-ray detection. They had achieved a value of accuracy of 96% using the ChestXray image dataset. Building on these existing approaches, this study leverages the qualities of transformer-based models, particularly MedViT and Swin Transformer, to address current limitations in lung classification and set a modern benchmark in diagnostic performance. Selvan et al. (19) had centered on the segmentation of lungs from the chest X-ray pictures. The proposed model has gotten the value of dice score of 0.8503 and an accuracy of 0.8815. Kim et al. (20) had performed lung segmentation using a self-attention module using a publicly available dataset of 138 images and had obtained the positive predictive value as 0.974. Vardhan et al. (21) has presented a framework using pre-trained models. They had performed validation with 286 images and had achieved a value of recall as 62.12% and average precision as 62.44%. Lascu et al. (22) had presented transfer learning-based models for the classification of COVID-19, Pneumonia, and healthy lungs. They had obtained the value of accuracy as 94.9%. Teixeira et al. (23) had performed training of the model using different CNN architectures. They had obtained the F1-score of 0.74.

Table 1 shows the findings obtained from the existing literature.

**TABLE 1:** Findings from existing literature.

References	Approach	Dataset/No. of images	Findings
Hage Chehade et al. (7)	CycleGAN	ChestX-ray 14/112,120	• Reduced electronic artifacts using CycleGAN pre-processing. • Integrated Attention mechanism to focus on relevant features.
Patel et al. (8)	Customized EfficientNet-B4 & XAI	CheXpert/941	• Integrated XAI with thresholding techniques. • EfficientNetB4 is used for feature extraction.
Mahamud et al. (24)	DenseNet201	Lung disease/10,000	• DenseNet201 combined with multiple XAI techniques for lung disease diagnosis. • Preprocessing techniques are applied to improve image clarity.
Chutia et al. (9)	DenseNet201	NIH chest X-ray/9,409	• A modified DenseNet201 model with channel attention blocks is used for the detection of lung disease. • A hybrid pooling layer is used to enhance feature learning.
Shamrat et al. (11)	MobileNetV2	ChestX-ray 14 / 112,120	• Created a MobileLungNetV2 that improves lung abnormality detection by improving feature extraction. • Utilized advanced preprocessing methods—Gaussian filtering for denoising, CLAHE for contrast improvement, and data augmentation—to progress image quality and address class imbalance. • Conducted comprehensive assessment utilizing numerous performance metrics and Grad-CAM visualizations.
Huy and Lin (15)	DenseNet	ChestX-ray 14/5,000	• Presented a novel deep learning design, CBAMWDNet, particularly planned for tuberculosis determination, accomplishing high classification performance with negligible increase in computational cost. • Emphasized the significance of high-quality and high-quantity datasets, selecting an ideal open dataset that enabled effective model training with negligible adjustments. • Illustrated the superiority of CBAMWDNet through comparative assessment against existing deep learning models within the medical imaging domain.
Singh et al. (17)	CNN	CXR/5,856	• Created a residual quaternion neural network for pneumonia detection utilizing the CXR dataset. • Improved the base architecture by integrating spatial and channel attention modules without modifying hyperparameters. • Conducted a comparative examination to assess the performance effect of attention mechanisms on pneumonia classification accuracy.

## 3 Proposed work

**FIGURE 2:**
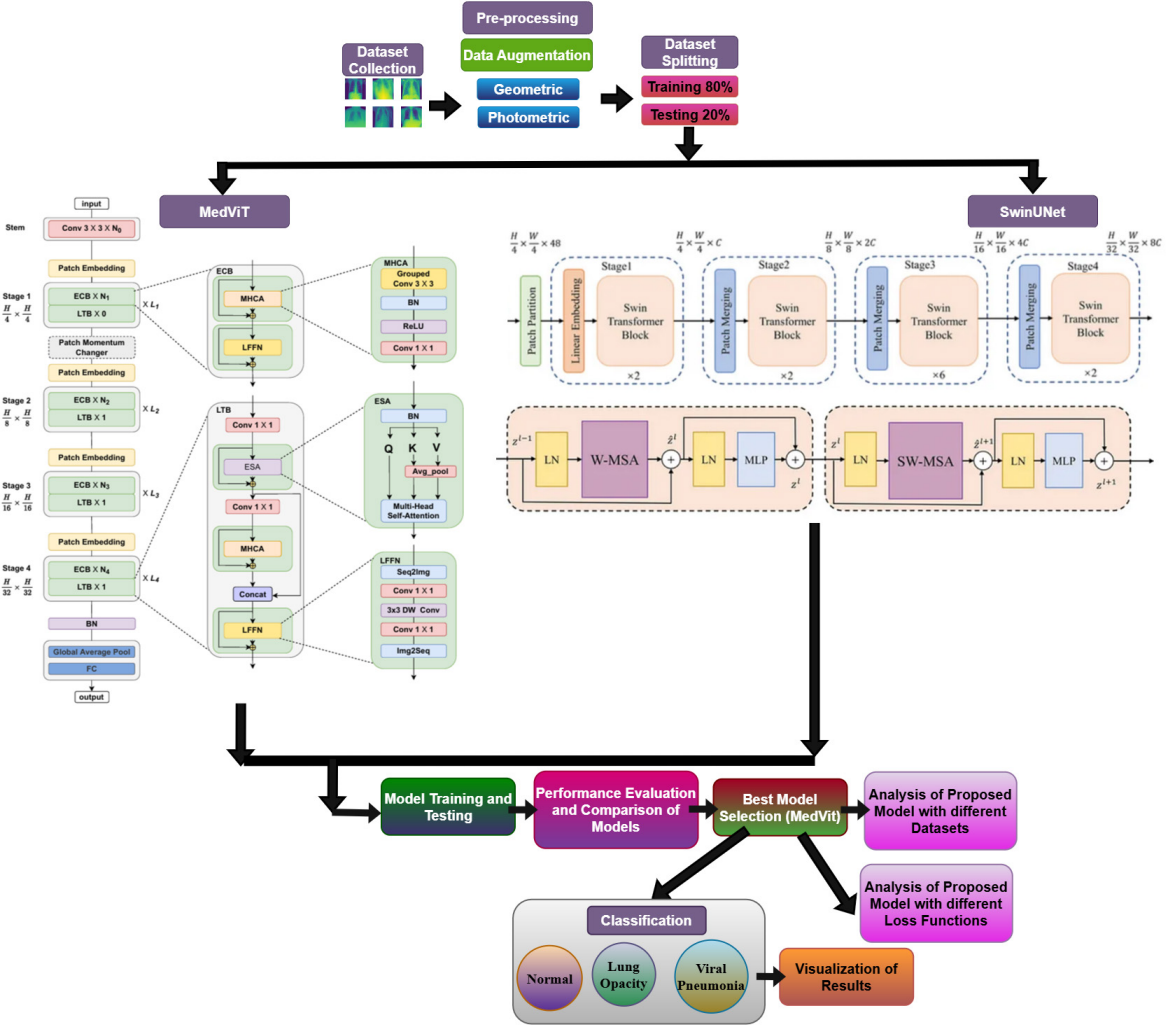
Proposed diagram of the study.

Figure 2 outlines the comprehensive technique utilized in a research study centered on the classification of chest X-ray images into categories such as Normal, Lung Opacity, and Viral Pneumonia. The workflow can be broadly separated into a few key stages: Pre-processing, Model Architecture (MedViT and SwinUNet), Model Training and Testing, Performance Assessment and Comparison, Model Selection, and finally, Analysis and Visualization of Results. The pre-processing stage includes preparing the raw chest X-ray dataset for subsequent modeling. It comprises three fundamental steps. The first step is the dataset collection, which gathers the essential chest X-ray images. The next step is data augmentation. The figure particularly notices “Geometric” and “Photometric” augmentations. The dataset splitting divided the collected and augmented dataset into two subsets: A training set of 80% utilized to train the models and a testing set of 20% utilized to assess their execution on unseen data. In addition to leveraging transformer-based models for precise lung infection classification, our proposed approach emphasizes information security by outlining the potential of security measures to guarantee the integrity and privacy of medical imaging information.

Further figure presents two distinct deep learning designs considered within the study: MedViT and SwinUNet. The MedViT design shows up to be a Vision Transformer-based model, particularly outlined for medical image analysis, and it features a progressive structure with different stages. SwinUNet design combines the strengths of the Swin Transformer and the U-Net. It moreover shows a multi-stage hierarchical design. MedViT is a hybrid deep learning design that combines convolutional layers with transformer blocks, empowering it to capture both local textures and global contextual features, making it especially successful for medical image analysis. Swin Transformer introduces a hierarchical vision transformer framework utilizing shifted windows, which improves computational efficiency and enables fine-grained feature extraction, proving beneficial for high-resolution medical imaging tasks like lung infection classification.

The models are trained utilizing the training dataset and subsequently, their performance is assessed utilizing the held-out testing dataset to evaluate their generalization ability. The results obtained are compared using important evaluation metrics. Based on the performance comparison, the best-performing model is selected. The figure shows that MedViT was chosen as the predominant model in this study. Further examination is conducted on the chosen best model on different datasets and testing with different loss functions during training to possibly optimize its performance further. The ultimate goal of the study is to precisely classify the chest X-ray images into the predefined categories: Normal, Lung Opacity, and Viral Pneumonia. At last, the study culminates in the classification of chest X-ray images and the visualization of the obtained results to provide a comprehensive understanding of the model’s capabilities and limitations in this critical medical imaging task.

### 3.1 Dataset description

The “Lung X-Ray Image Dataset” is an inclusive assortment of X-ray images that plays a noteworthy part in the discovery and determination of lung diseases (25). The dataset covers many high-quality X-ray images, methodically collected from varied sources, including hospitals, clinics, and healthcare institutions. The dataset comprises 3,475 X-ray images. The distinctive classes include the Normal class comprising 1,250 images. Lung Opacity class comprises 1,125 images. Viral Pneumonia class comprises 1,100 images related to viral pneumonia cases, contributing to the understanding and recognizable proof of this specific lung disease. Table 2 illustrates the disease-class-wise images.

**TABLE 2:** Disease class-wise image samples.

Disease name	Disease class	Image count	Image sample 1	Image sample 2	Image sample 3
Normal	0	1,250	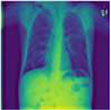	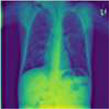	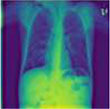
Lung opacity	1	1,125	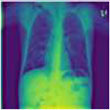	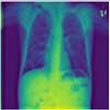	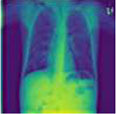
Viral pneumonia	2	1,100	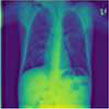	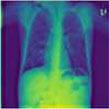	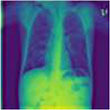

### 3.2 Data augmentation

The input images are augmented with different augmentation techniques (26). The data augmentation techniques are used to increase the total count of images in the data folder so that training can be performed on the maximum images. The different geometric and photometric augmentation techniques are used in the study. The common geometric augmentation techniques include rotation, flipping, scaling (with zoom in/out), shifting, shearing, cropping and padding. Whereas photometric augmentation includes brightness and contrast adjustment, gamma correction, Gaussian noise and blurring. The dataset was first divided into training, and testing subsets, and data augmentation was applied exclusively to the training set to prevent data leakage and ensure independence across splits. Augmentation was conducted only on the training set, ensuring that no augmented or near-duplicate images appeared in either the validation or testing sets. To address the concern of overfiting, we have incorporated additional evaluation and analysis to demonstrate that the model learns meaningful and discriminative features rather than memorizing synthetic variations.

a. *Rotation*: The rotation transforms an image by rotating it by an angle of 20 degrees. A pixel location of (a, b) in the original image with its new coordinates as (a’, b’) after performing rotation. The eq. for the same is given in (1):
$$\left[\begin{array}{c} a^{\prime }\\ b^{\prime } \end{array}\right]=\left[\begin{array}{cc} \cos\theta & -\sin\theta \\ \sin\theta & \cos\theta \end{array}\right]\,\left[\begin{array}{c} a\\ b \end{array}\right]$$ (1)
b. *Shifting*: This operation involves a width and height shift that moves image horizontally and vertically. The transformation of each pixel coordinate is given in Equations (2) and (3):
$$p^{{}^{\prime }}=p+w_{s}.W,q^{{}^{\prime }}=q$$ (2)
$$a^{{}^{\prime }}=a+h_{s}.H,b^{{}^{\prime }}=b$$ (3)
c. *Zooming*: This operation scales the image by a factor Z with its center c_*a*_ and c_*b*_. As shown in Equation (4)
$$a^{{}^{\prime }}=c_{a}+Z\,\left(a-c_{a}\right),b^{{}^{\prime }}=c_{b}+Z\,\left(b-c_{b}\right)$$ (4)
d. *Flipping*: This operation can be performed horizontally and vertically, both as shown in Equations (5) and (6).
$$a^{\prime }=H-a,b^{\prime }=b$$ (5)
$$b^{\prime }=V-b,a^{\prime }=a$$ (6)
  These operations improve the robustness of the model.

Although augmentation was applied proportionally to increase the number of samples in each class. To address this, patient-level independence was ensured during dataset splitting and used balanced mini-batch sampling during training to prevent model bias toward majority classes.

#### 3.2.1 Dataset splitting

**FIGURE 3:**
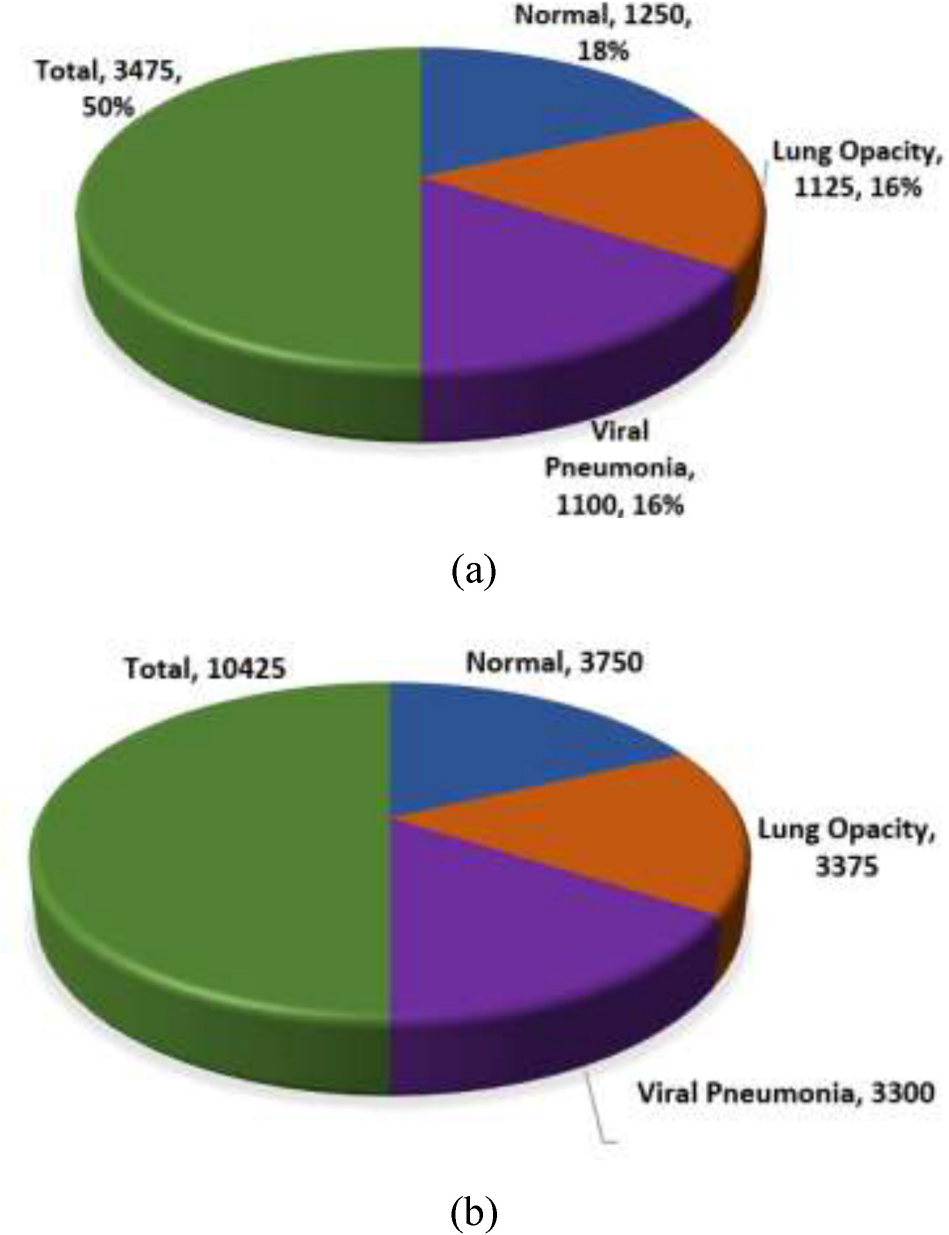
Splitting of the dataset **(a)** before augmentation, **(b)** after augmentation.

The dataset used for training purposes is taken from the Kaggle repository. Earlier, the total count of lung disease images was 3,475, whereas after the augmentation techniques application the count of images increased to 10,425, as shown in Table 3. The splitting ratio used is 80:20 i.e., with the ratio of training as 80% and testing as 20%. Figure 3 shows the splitting of a dataset into training and testing.

**TABLE 3:** Image count before and after augmentation.

Disease name	Total count before augmentation	Total count after augmentation	Training (80%)	Testing (20%)
Normal	1,250	3,750	3,000	750
Lung opacity	1,125	3,375	2,700	675
Viral pneumonia	1,100	3,300	2,640	660
Total	3,475	10,425	8,340	2,085

### 3.3 Model architectures

This section uses two architectures, namely MedViT V2 and Swin Transformer. Both models are trained on the Lung X-Ray Image Dataset.

#### 3.3.1 MedFormer/MedViT model

**FIGURE 4:**
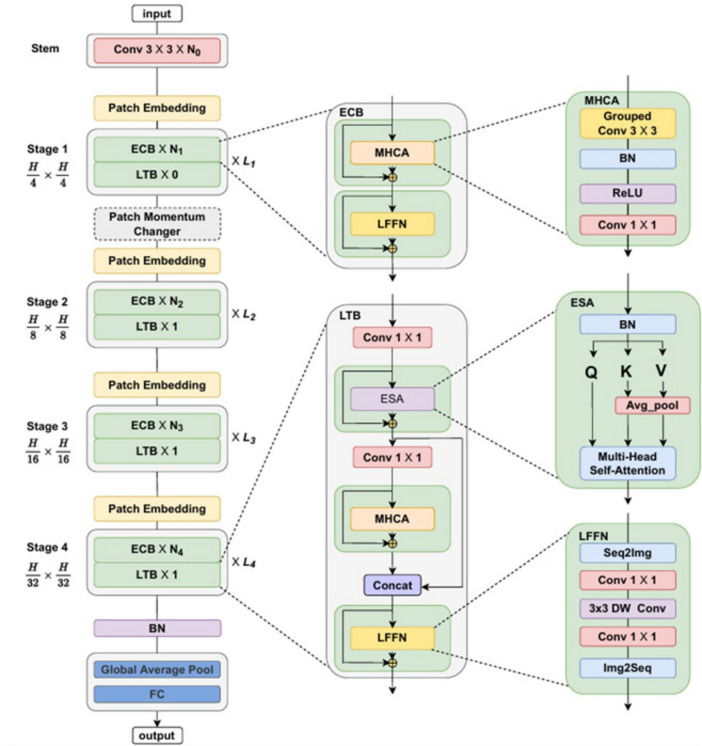
MedViT model architecture (27).

MedViT coordinates the qualities of Convolutional Neural Networks (CNNs) and Vision Transformers (ViTs) to viably capture both local and global features in medical images, as shown in Figure 4. This hybrid approach addresses challenges like data shortage, domain shifts, and adversarial robustness. This design is especially successful for medical images such as chest X-rays, where both fine-grained local details and long-range relevant data are critical for the accurate conclusion. MedViT integrates convolutional blocks at shallow layers and transformer blocks at deeper layers. This hybrid plan improves the model’s ability to capture both local low-level features and global high-level semantics.


O=T⁢r⁢a⁢n⁢s⁢f⁢o⁢r⁢m⁢e⁢r⁢_⁢B⁢l⁢o⁢c⁢k⁢(C⁢o⁢n⁢v⁢S⁢t⁢e⁢m⁢(X)) (7)

Here, *O* is the output, *X* is the input image, shallow features are extracted by *ConvStem* and *Transformer*_*Block* captures long-range dependencies.

The MedViT design is composed of a few key components planned to viably handle medical images by combining convolutional and transformer-based methods. It starts with the Patch Embedding Layer, which partitions the input image into smaller patches and embeds them into a higher-dimensional space suitable for transformer processing. Following this, the Efficient Convolution Block (ECB) extricates local features through convolutional operations while maintaining spatial hierarchies essential for recognizing fine-grained medical details. The Local Transformer Block (LTB) applies self-attention mechanisms inside localized regions, capturing long-range conditions in a computationally proficient way. To further improve the feature representations, the Transformer Augmentation Block (TAB) joins global context by leveraging deeper transformer layers. In conclusion, MedViT follows a Hierarchical Structure, organizing the network into progressive stages that systematically reduce spatial dimensions while expanding the depth and complexity of the extracted features, empowering strong and versatile learning for medical image classification tasks.

#### 3.3.2 Swin transformer

**FIGURE 5:**
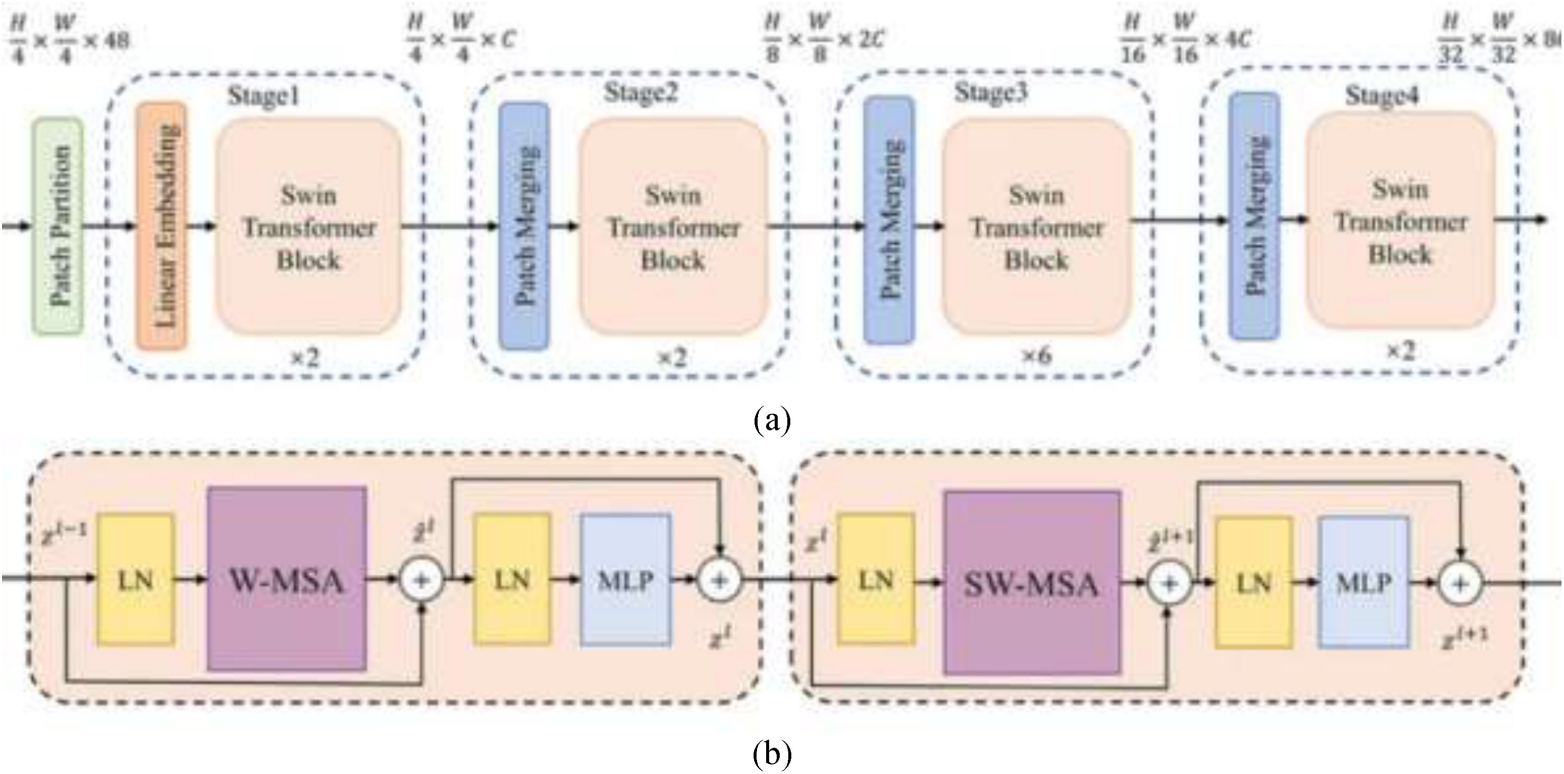
(a) Swin transformer, **(b)** swin transformer block (28).

Swin Transformer or Shifted Window Transformer is a hierarchical vision transformer that forms images in a local windowed way while also enabling global feature interaction through an intelligent window-shifting mechanism, as shown in Figure 5. The Swin Transformer design presents a set of carefully planned components tailored for effective and versatile image representation. It begins with Patch Partitioning, where the input image is separated into fixed-size non-overlapping patches (e.g., 4 × 4), each of which is then flattened and passed through a linear layer in the Patch Embedding stage to produce token embeddings. To enable learning at multiple scales, the model develops a Hierarchical Representation by progressively decreasing the spatial resolution and expanding the feature dimensionality over stages, forming a feature pyramid. The attention mechanism in Swin Transformer is based on Window-based Multi-head Self Attention (W-MSA), where self-attention is computed inside local non-overlapping windows, essentially lessening computational overhead. To upgrade the model’s ability to capture global context and empower cross-window connections, the Shifted Window-based Self-Attention (SW-MSA) is introduced in alternating layers, shifting the window positions to overlap with adjoining regions. Following the attention layers, each block incorporates a Multi-Layer Perceptron (MLP) composed of fully connected layers, GELU activations, and normalization layers to refine the learned features. All through the network, Layer Normalization and Residual Connections are applied around both attention and MLP blocks to improve training stability and model convergence. This combination of local and global attention, hierarchical structure, and stable training design makes the Swin Transformer highly viable for visual recognition tasks, including medical image classification.

### 3.4 Hyperparameter tuning using loss function

Hyperparameter tuning (29) is crucial in optimizing the performance of deep learning models. Choosing a suitable loss function, which establishes the model’s training success metric, is a crucial step in this tuning process. The loss function and other hyperparameter selections have a big influence on the performance of a deep learning model. Different loss functions are suited for different types of problems (e.g., regression, classification) and can affect how the model learns from the data. By tuning hyperparameters related to loss functions, such as the learning rate or the weight associated with certain classes, reduce overfitting and ultimately increase the model’s performance metrics. In the framework of classification tasks, the loss functions that are used in this research include Binary Cross-entropy, Hinge Loss, and Kullback-Leibler (KL) Divergence.

- *Binary cross-entropy:* utilized in binary classification assignments and processes the distinction between two probability distributions: the predicted likelihood distribution output by the model and the genuine distribution. The formula for binary cross-entropy is given in Eq. (8):

$$\text{B}inary\text{C}rossentropy=-\frac{1}{\text{N}}\sum_{i=1}^{N}[{\text{y}}_{i}\cdot \text{l}og\left(\hat{{\text{y}}_{i}}\right)+\left(1-{\text{y}}_{i}\right)\cdot$$ (8)

$$\text{l}og\left(1-\hat{{\text{y}}_{i}}\right)]$$

Where N is the number of samples, y_*i*_ is the true label for sample i (0 or 1), $\left(\hat{{\text{y}}_{i}}\right)$ is the predicted probability that sample i belongs to class 1. The formula penalizes incorrect predictions more strongly when the model is confident about its incorrect predictions.

- *Hinge loss*: is commonly used in binary classification tasks, particularly in Support Vector Machine (SVM) models. It maximizes the margin between classes. The hinge loss is defined in Eq. (9):

$$\text{H}\,i\,n\,g\,e\,\text{L}\,o\,s\,s=\text{m}\,a\,x\,\left(0,1-{\text{y}}_{t\,r\,u\,e}*{\text{y}}_{p\,r\,e\,d}\right)$$ (9)

Where, y_*true*_ is the true label (either -1 or 1 in binary classification), y_*pred*_ is the predicted value (before applying any activation function). The max function ensures that the loss is 0 when the prediction is correct and positive when the prediction is incorrect.

- *Kullback-Leibler (KL) divergence*: is often used in the context of comparing two probability distributions, such as a predicted distribution and a true distribution. The formula for KL divergence is shown in Eq. (10):

$$D_{K\,L}\left(P||Q\right)=\sum_{i}P\left(i\right)\cdot \log\left(\frac{P\,\left(i\right)}{Q\,\left(i\right)}\right)$$ (10)

In essence, hyperparameter tuning using loss functions is performed to enhance the model’s performance and achieve the best possible results for a given task and dataset. These loss functions were chosen based on their appropriateness for distinctive classification scenarios, and their effect on model execution will be efficiently assessed to determine the most effective approach for the given task.

## 4 Results

In this segment, distinctive types of analysis are performed for the classification of lung disease using X-ray images. In addition to accomplishing high classification accuracy, our approach highlights the potential of joining security mechanisms to defend model outputs and understanding information, strengthening the unwavering quality of AI-driven diagnostic tools in delicate clinical situations. Ablation analysis is performed utilizing different designs, followed by examination with different loss functions that include comparing their classification results. Qualitative analysis involves visually inspecting classified images to assess their quality and identifying areas for change. State-of-the-art comparison includes comparing the proposed models’ execution with different methods. The experiments were conducted on a workstation configured with an Intel Core i5 processor, Intel Iris integrated graphics, and 16 GB RAM. The proposed model demonstrated an average inference time of approximately 65 ms per image, allowing for rapid classification and supporting near real-time diagnostic applications. This performance indicates that the model can be deployed effectively in clinical or telemedicine settings where timely decision-making is essential. The hyperparameters in model were selected through systematic manual tuning. Iterative experimentation is performed on the validation set by varying the learning rate, Adam optimizer (β^1^ = 0.9, β^2^ = 0.999) was selected based on smoother gradient updates compared to SGD and RMSProp.

### 4.1 Ablation analysis

In this section, ablation analysis is performed using two different architectures, namely MedViT and Swin Transformer.

**FIGURE 6:**
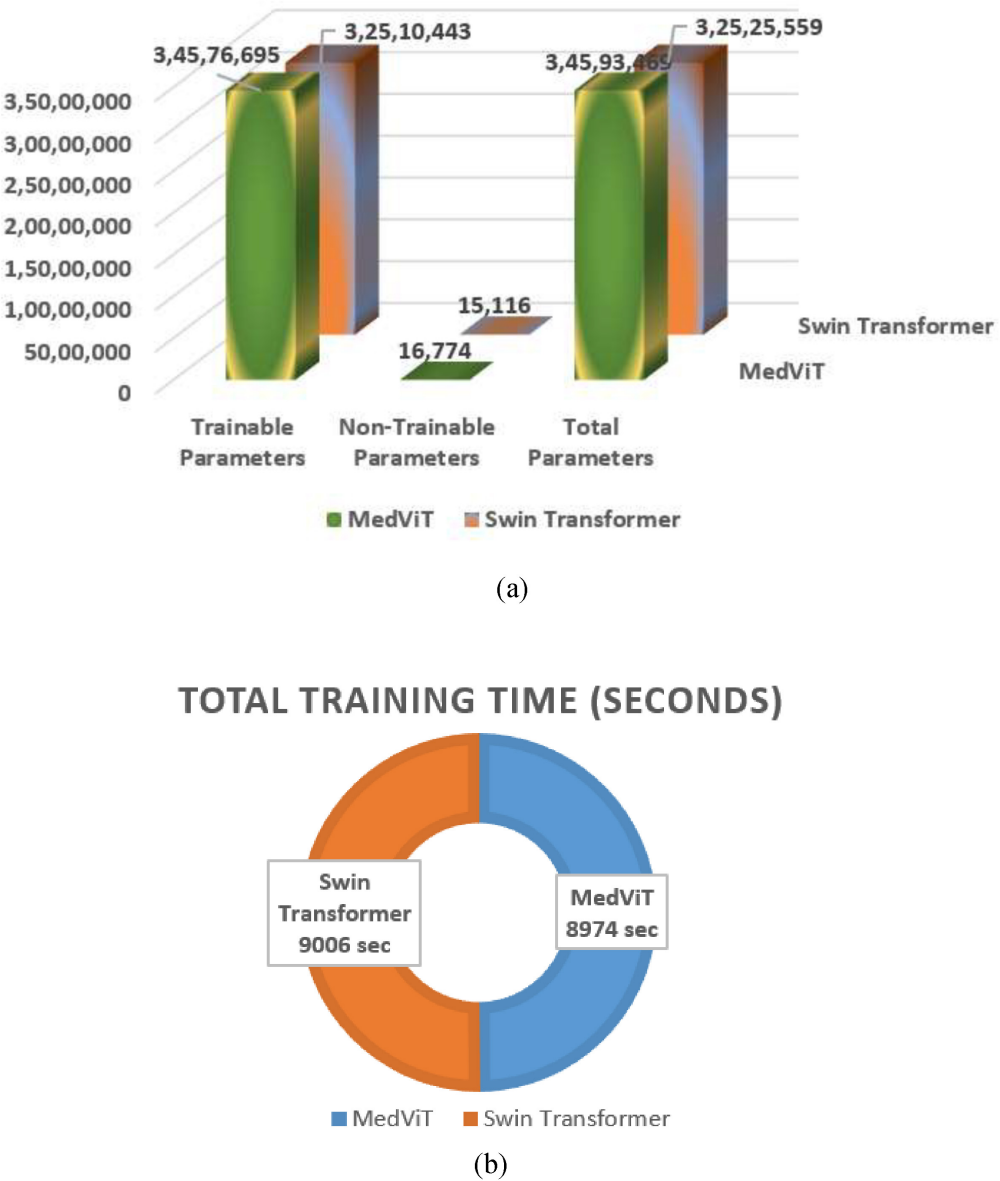
Ablation analysis **(a)** parameter status, **(b)** time status.

The computational complexity and proficiency of the models were evaluated based on the number of parameters and training time, as illustrated in Table 4 and Figure 6.

**TABLE 4:** Training time and parameter count of different architectures.

	Trainable parameters	Non-trainable parameters	Total parameters	Total training time (seconds)
MedViT	34,576,695	16,774	34,593,469	8,974
Swin transformer	32,510,443	15,116	32,525,559	9,006

The MedViT model comprised around 34.6 million total parameters, with 34.57 million trainable parameters, and required 8,974 s for total training. In comparison, the SwinUNet model had a slightly lower parameter number, totaling 32.53 million, with 32.51 million trainable parameters, but took hardly longer to train at 9,006 s. These results recommend that whereas MedViT contains a higher parameter count, it accomplishes comparable or superior training proficiency, showing its compelling design for taking care of lung disease classification tasks. MedViT combines convolutional layers with transformer blocks, enabling both local feature extraction and global context modeling. Figure 6 shows the parameters status and time status of MedViT and Swin Transformer.

**FIGURE 7:**
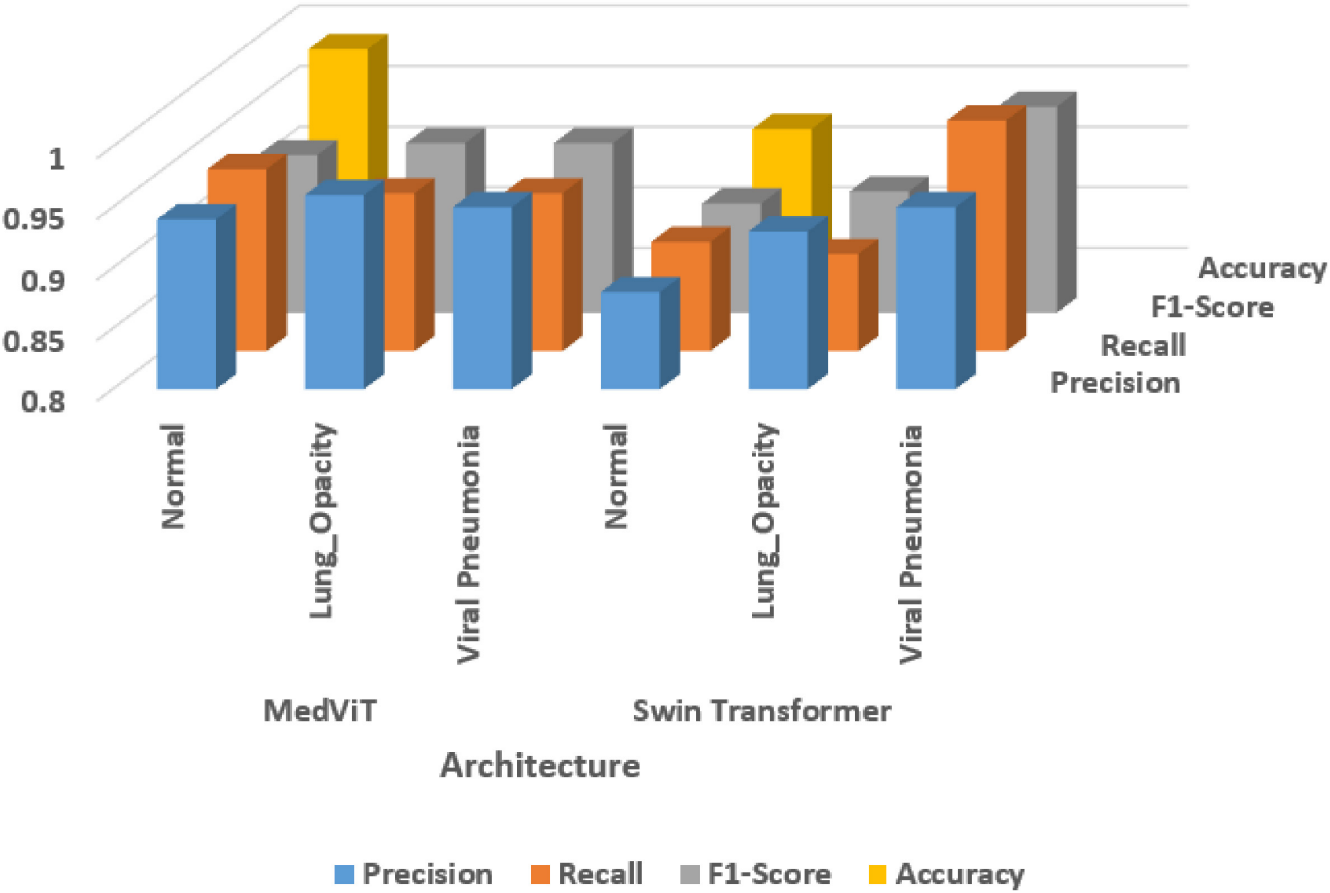
Performance analysis using evaluation metrics.

Table 5 and Figure 7 shows the performance analysis using different metrics. It compares the performance of two deep learning architectures—MedViT and Swin Transformer—across three disease classes: Normal, Lung Opacity, and Viral Pneumonia.

**TABLE 5:** Performance parameter analysis of both architectures.

Architectures	Disease class	Precision	Recall	F1-Score	Accuracy	Loss
MedViT	Normal	0.94	0.95	0.93	0.986	0.09
	Lung_opacity	0.96	0.93	0.94		
	Viral pneumonia	0.95	0.93	0.94		
Swin transformer	Normal	0.88	0.89	0.89	0.92	0.16
	Lung_opacity	0.93	0.88	0.90		
	Viral pneumonia	0.95	0.99	0.97		

Overall, MedViT beats the Swin Transformer in all assessed metrics, including accuracy, recall, F1-score, precision, and loss. For the Normal class, MedViT accomplishes precision value as 0.94, a recall as 0.95, and an F1-score of 0.93, with an overall accuracy of 0.986 and a low loss of 0.09.

In the Lung Opacity category, MedViT again appears as predominant, getting a precision of 0.96, a recall of 0.93, and an F1-score of 0.94. For Viral Pneumonia, both models perform emphatically, but MedViT keeps up a slight edge with precision and recall both at 0.95 and 0.93 separately, leading to an F1-score of 0.94. The Swin Transformer excels in recall (0.99) and accomplishes the highest F1-score (0.97) among all sections, but its precision is marginally lower at 0.95. In summary, MedViT reliably illustrates more balanced and higher execution over all disease classes and evaluation metrics, making it the more successful model in general for the classification tasks in this study. Table 6 shows the comparison of MedViT and Swin Transformer on different criteria.

**TABLE 6:** Comparison of both models on different criteria.

Criterion	MedViT	Swin transformer
Architecture type	CNN and vision transformer combination	Windowed self-attention
Local feature extraction	Efficient convolution block maintains spatial hierarchies	Window-based multi-head self-attention
Global feature modeling	Transformer augmentation block	Sifted window attention
Data efficiency	High	Moderate
Robustness to noise	Very strong	Moderate
Ease of training	Medium	Complex
Computational efficiency	Medium	Medium
Explainability tools compatibility	Performs well due to the CNN backbone and hybrid design	Effective but slightly less localized feature attribution

### 4.2 Analysis with different loss functions

**FIGURE 8:**
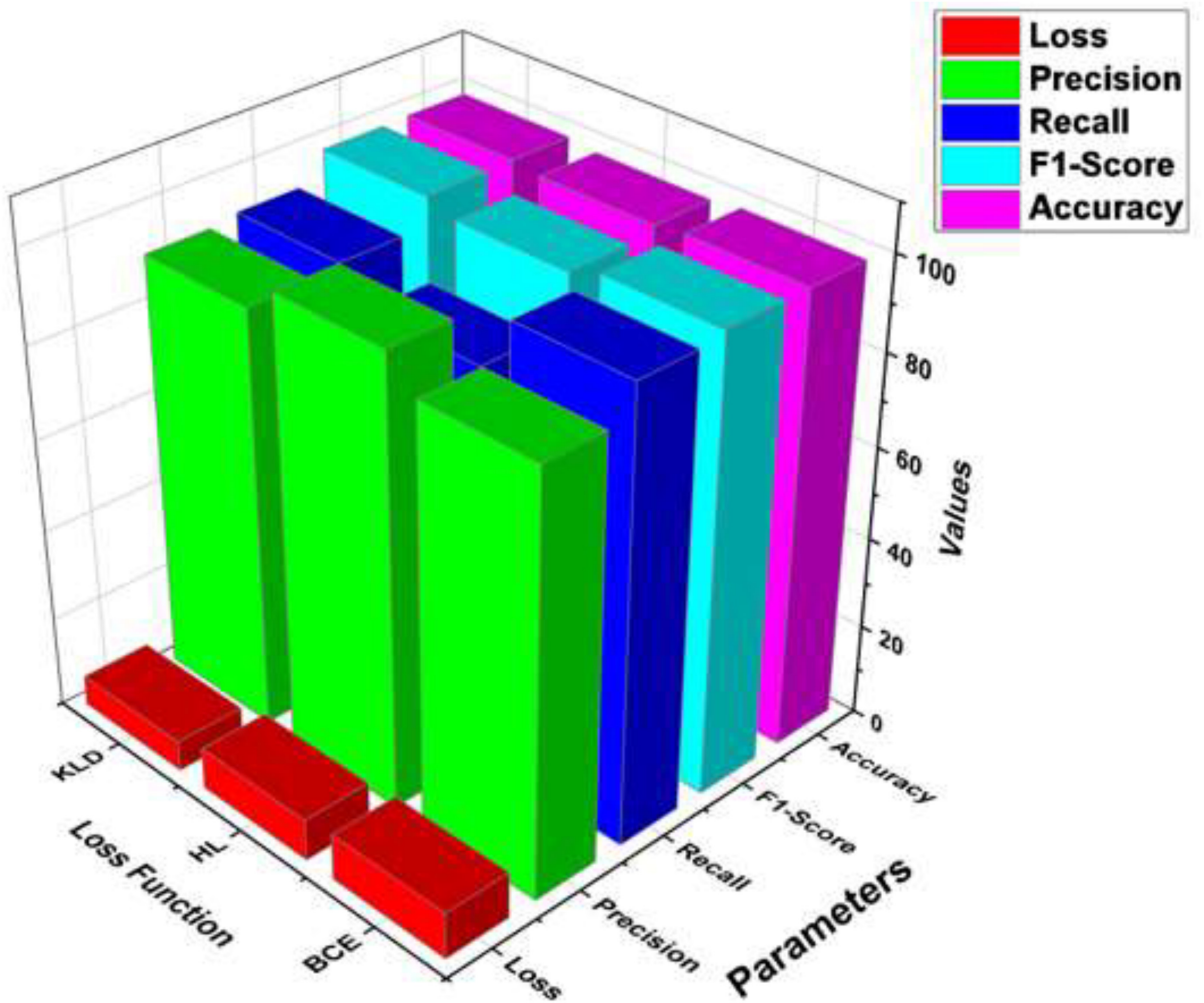
Loss function analysis on MedVit model.

Table 7 and Figure 8 compare the execution of three loss functions—Binary Cross-Entropy (BCE), Hinge Loss (HL), and Kullback-Leibler Divergence (KLD)—based on key assessment metrics: precision, F1-score, recall, accuracy, and loss. Hinge Loss illustrates the highest precision at 97.10%, demonstrating strong execution in accurately recognizing positive cases. However, it has the lowest recall at 85.32%, recommending that it misses a noteworthy number of actual positives. Its F1-score (95.93%) and accuracy (97.60%) are competitive, and it keeps up a moderately low loss of 9.14.

**TABLE 7:** Analysis of MedViT architecture with different loss functions.

Loss function	Precision	Recall	F1-Score	Accuracy	Loss
Binary cross-entropy	90.67	97.34	98.45	98.43	10.23
Hinge loss	97.10	85.32	95.93	97.60	9.14
Kullback-leibler (KL) divergence	91.10	92.25	98.23	98.50	6.67

Binary Cross-Entropy offers the highest recall at 97.34%, making it successful at capturing most positive instances. It too yields the highest F1-score at 98.45%, indicating a strong balance between precision and recall. Its precision (90.67%) and accuracy (98.43%) are slightly lower, and it has the highest loss value at 10.23, demonstrating less effectiveness in error minimization.

KL Divergence strikes a balance between the two, with consistent execution over all metrics. It accomplishes a precision of 91.10%, recall of 92.25%, and a strong F1-score of 98.23%. It also records the highest accuracy at 98.50% and the lowest loss at 6.67, recommending it is the most effective in decreasing training error while keeping up strong classification performance.

In summary, while each loss function has its qualities, KL Divergence offers the best overall trade-off between execution and training effectiveness, making it a favorable choice for optimizing deep learning models in this context. KL Divergence measures the difference between the predicted and true probability distributions, allowing the model to assign higher sensitivity to minority classes. This helps prevent the model from being biased toward majority classes and improves balanced learning across all categories.

### 4.3 Analysis with different datasets

**FIGURE 9:**
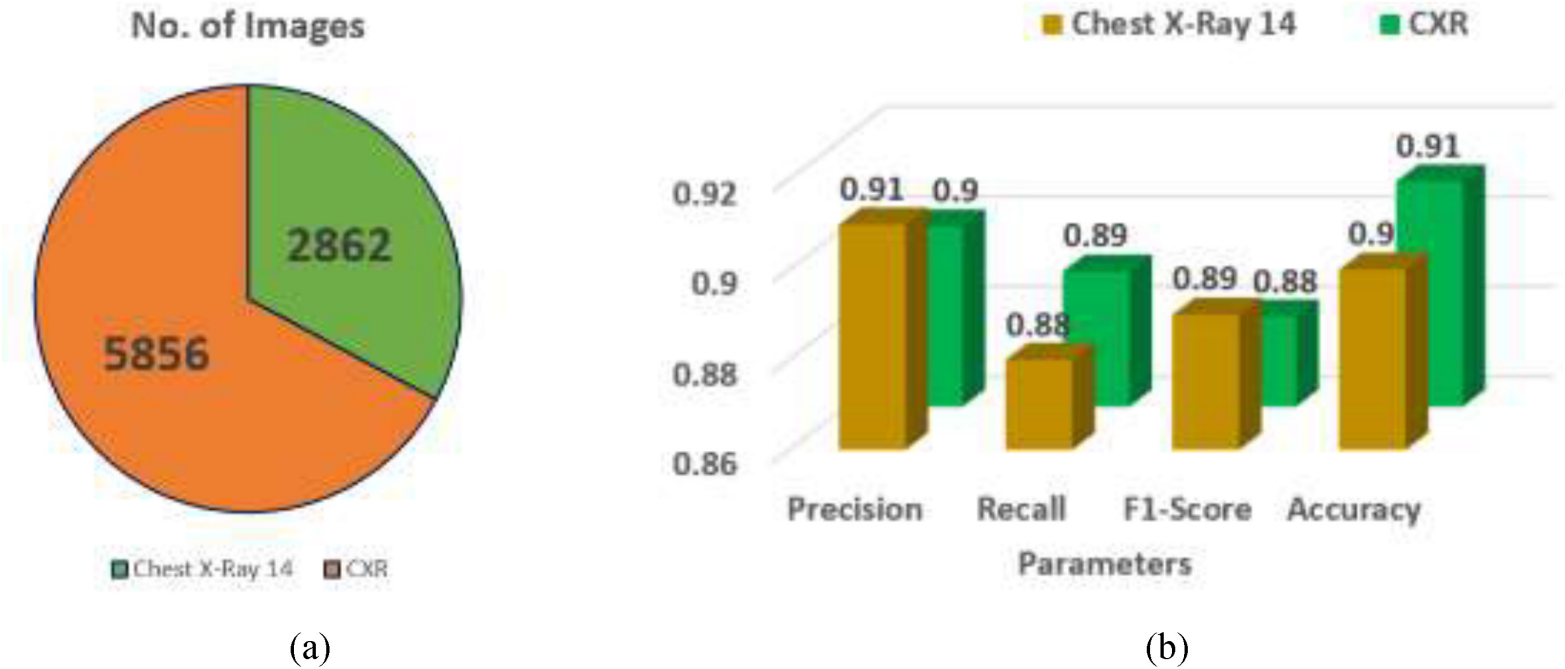
Dataset analysis **(a)** image count, **(b)** parameters.

The other two datasets that are taken for comparison are Chest X-Ray 14 and CXR datasets, with 2,862 and 5,856 X-ray images, respectively. The dataset ChestX-ray14 contains 112,120 chest X-ray images and only 2,862, X-ray images were chosen from the dataset. Table 8 and Figure 9 show the MedViT architecture analysis with different datasets.

**TABLE 8:** Analysis of MedViT architecture with different datasets.

Dataset name	No. of images	Precision	Recall	F1-score	Accuracy
Chest X-ray 14 (30)	2,862	0.91	0.88	0.89	0.90
CXR (31)	5,856	0.90	0.89	0.88	0.91

The execution of two chest X-ray datasets, Chest X-Ray 14 and CXR, was assessed based on key classification metrics. The Chest X-Ray 14 dataset incorporates 2,862 images and attained a precision as 0.91, a recall as 0.88, a F1-score of 0.89, and an accuracy of 0.90. In comparison, the CXR dataset contains a larger number of images (5,856) and recorded a marginally lower precision of 0.90, a better recall of 0.89, an F1-score of 0.88, and the most elevated accuracy at 0.91.

In general, Chest X-Ray 14 illustrates somewhat better precision and F1-score, showing more grounded performance in minimizing false positives and keeping up a balanced trade-off between precision and recall. On the other hand, the CXR dataset performs way better in terms of recall and accuracy, suggesting it is more viable at capturing true positive cases and accomplishing more correct expectations overall. The choice between the two datasets should be guided by the specific objectives of the diagnostic task—for instance, prioritizing precision and balanced execution with Chest X-Ray 14 or maximizing detection and overall correctness with the CXR dataset.

### 4.4 Analysis with different models

**FIGURE 10:**
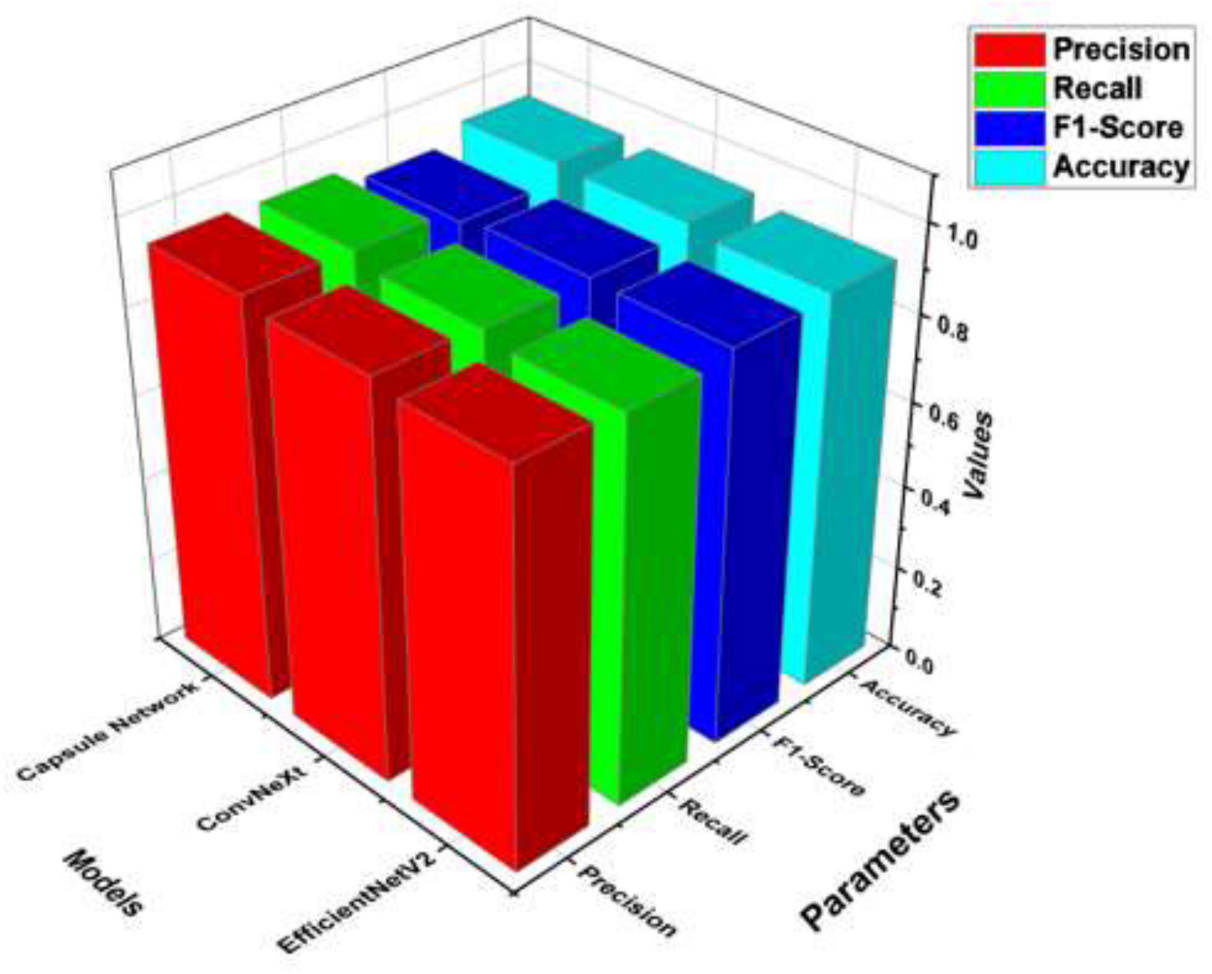
Metrics comparison of different models.

Table 9 and Figure 10 present a comparative assessment of three deep learning models—EfficientNetV2, ConvNeXt, and Capsule Network—based on four key execution parameters: Precision, Recall, F1-Score, and Accuracy. EfficientNetV2 illustrates solid overall execution with an accuracy of 92%. It keeps up a balanced trade-off between precision (0.905) and recall (0.89), resulting in an F1-score of 0.91. This demonstrates solid but marginally less ideal classification performance compared to the other models. ConvNeXt beats EfficientNetV2 in all metrics, accomplishing the highest F1-score (0.915) among the three, as well as improved precision (0.922) and recall (0.91). It also shows the highest accuracy (93.5%), reflecting a well-rounded and strong performance over the board.

**TABLE 9:** Analysis with different models.

Models	Precision	Recall	F1-score	Accuracy
EfficientNetV2	0.905	0.89	0.91	0.92
ConvNeXt	0.922	0.91	0.915	0.935
Capsule network	0.942	0.936	0.901	0.94

Capsule Network excels especially in precision (0.942) and recall (0.936), outperforming both EfficientNetV2 and ConvNeXt in these areas. However, its F1-score (0.901) is somewhat lower than ConvNeXt’s, proposing a minor imbalance in its precision-recall trade-off. Despite this, it still keeps up a high overall accuracy of 0.94.

While all three models display solid performance, Capsule Network leads in precision and recall, making it exceedingly successful in accurately recognizing both positive and negative cases. ConvNeXt, however, accomplishes the best balance over all metrics, especially in terms of the F1-score and overall accuracy, demonstrating it may be the most reliable model in common applications. EfficientNetV2, though slightly behind the others, still offers strong execution and may be preferred in resource-constrained situations due to architectural efficiency.

### 4.5 Inference and model performance analysis with Grad-CAM

**FIGURE 11:**
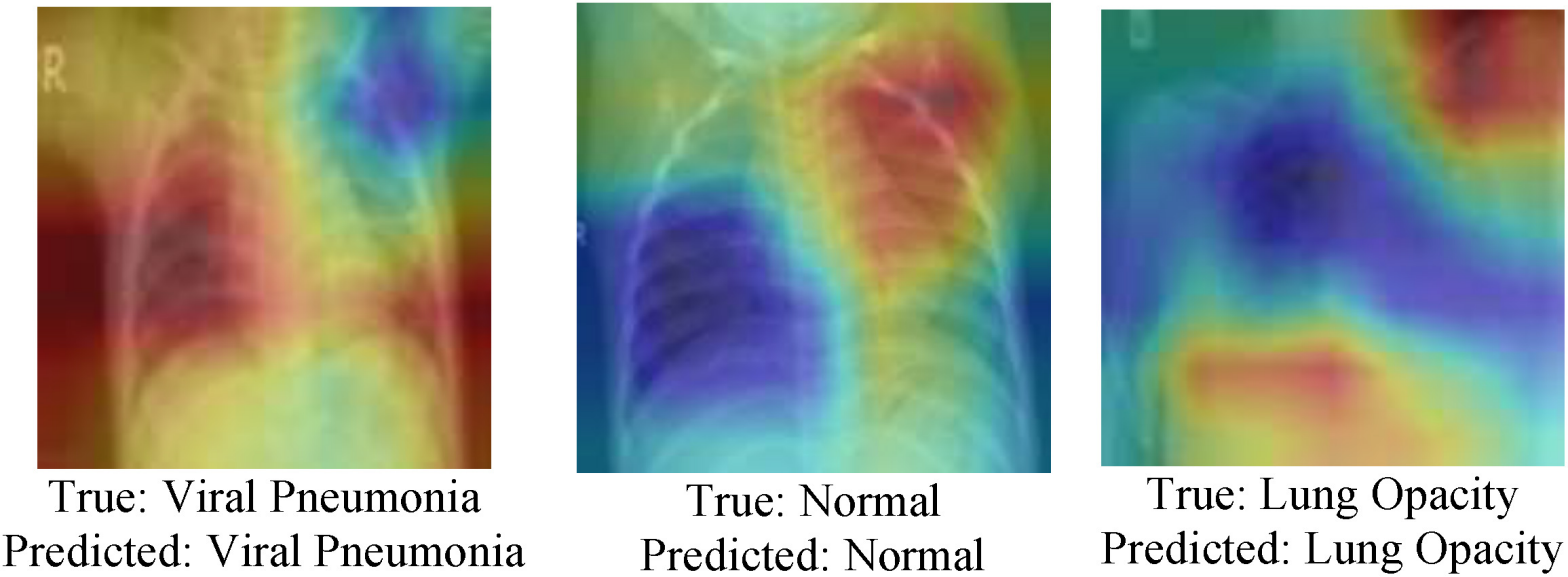
Grad-CAM inference.

Gradient-weighted Class Activation Mapping (Grad-CAM) is a predominant visualization method utilized to advance the interpretability of convolutional neural networks (CNNs) and hybrid designs (32). It makes class-specific heatmaps by utilizing the gradients of the target class flowing into the final convolutional layer, highlighting the basic regions inside the input image that most strongly affect the model’s expectation. Inside the context of medical AI, particularly in medical image classification tasks, Grad-CAM serves a pivotal part by providing visual clarifications of the model’s decision-making process. Usually it is important in high-stakes domains like radiology, where the results of incorrect diagnoses can be extreme. By overlaying attention maps on medical images such as chest X-rays, Grad-CAM permits clinicians and analysts to confirm whether the model is focusing on medically significant anatomical structures, such as areas with opacities, consolidations, or irregular designs. The Grad-CAM inference is shown in Figure 11.

In this study, Grad-CAM is utilized to visualize and compare the regions of interest recognized by both the MedViT and Swin Transformer models during the classification of lung diseases. These visualizations provide profitable insights into the internal workings of the models, helping to evaluate model reliability, feature localization accuracy, and the extent to which the models align with clinical reasoning. Grad-CAM in this manner improves the dependability and transparency of deep learning-based diagnostic devices and bolsters their potential integration into real-world medical practice.

### 4.6 Qualitative analysis

**FIGURE 12:**
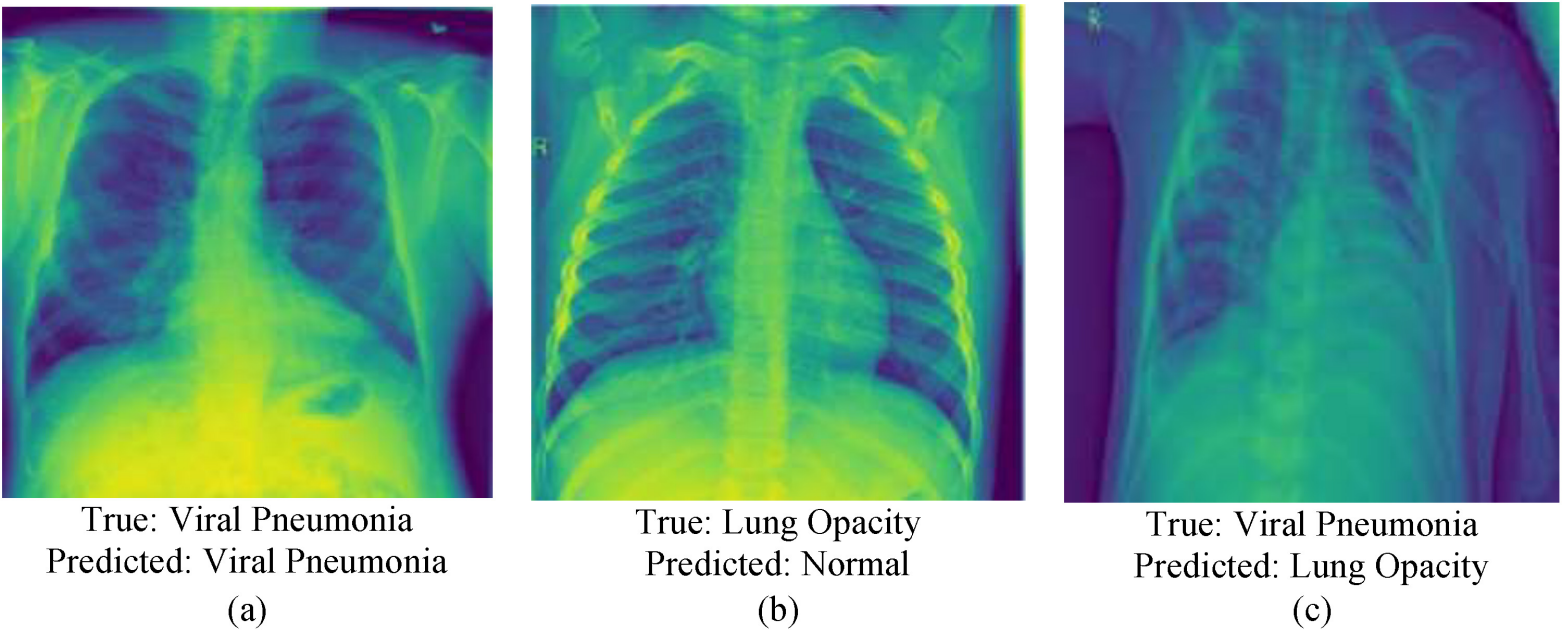
Qualitative analysis of chest X-Ray images **(a)** correctly classified, **(b,c)** misclassification.

To further assess the execution of the proposed model, a qualitative examination was conducted utilizing visualizations of Chest X-ray images, highlighting both correct classifications and misclassifications. Figure 12 presents representative cases from the test set. Figure 12a outlines a correctly classified case, where the ground truth was Viral Pneumonia, and the model precisely anticipated Viral Pneumonia, illustrating the model’s ability to capture relevant pathological features. In contrast, Figure 12b represents a misclassification, where the true label was Lung Opacity, but the model predicted Normal, demonstrating a potential challenge in recognizing subtle opacities from healthy lung structures. Additionally, Figure 12c shows another misclassified case, where the actual condition was Viral Pneumonia, but the model inaccurately predicted Lung Opacity, recommending possible feature overlap or uncertainty between these two classes. These visualizations give insight into the model’s decision-making process and highlight areas for further enhancement, especially in distinguishing between diseases with similar radiographic appearances.

The variability in classification performance can be attributed to a few components related to the complexity of chest X-ray images and the inherent challenges in medical image investigation. Correct classifications, such as the case in Figure 12a typically happen when the pathological features are noticeable and well-represented within the training data. In these cases, the model can successfully learn and generalize the visual designs related to particular conditions, such as distinct consolidations or infiltrates in Viral Pneumonia. On the other hand, misclassifications emerge due to numerous contributing factors. In Figure 12b, where Lung Opacity was inaccurately anticipated as Normal, the likely cause is the subtlety or localized nature of the opacity, which may closely resemble normal tissue designs, particularly in lower contrast. This could lead the model to miss minor anomalies. Furthermore, overlapping visual characteristics among infection categories—for instance, between Viral Pneumonia and Lung Opacity as seen in Figure 12c—can confuse the model. Both conditions may show with diffuse opacities or patchy penetrates, making it challenging to draw clear boundaries between them, especially in the absence of accompanying clinical data.

Moreover, class imbalance, limited annotated samples for certain illnesses, or noise and artifacts within the X-ray images can affect the model’s learning and generalization. These variables collectively contribute to the observed misclassification and emphasize the need for more diverse training data, improved feature extraction methods, and possibly multimodal approaches that coordinate clinical metadata for improved diagnostic precision.

### 4.7 Benchmarking against current approaches

**FIGURE 13:**
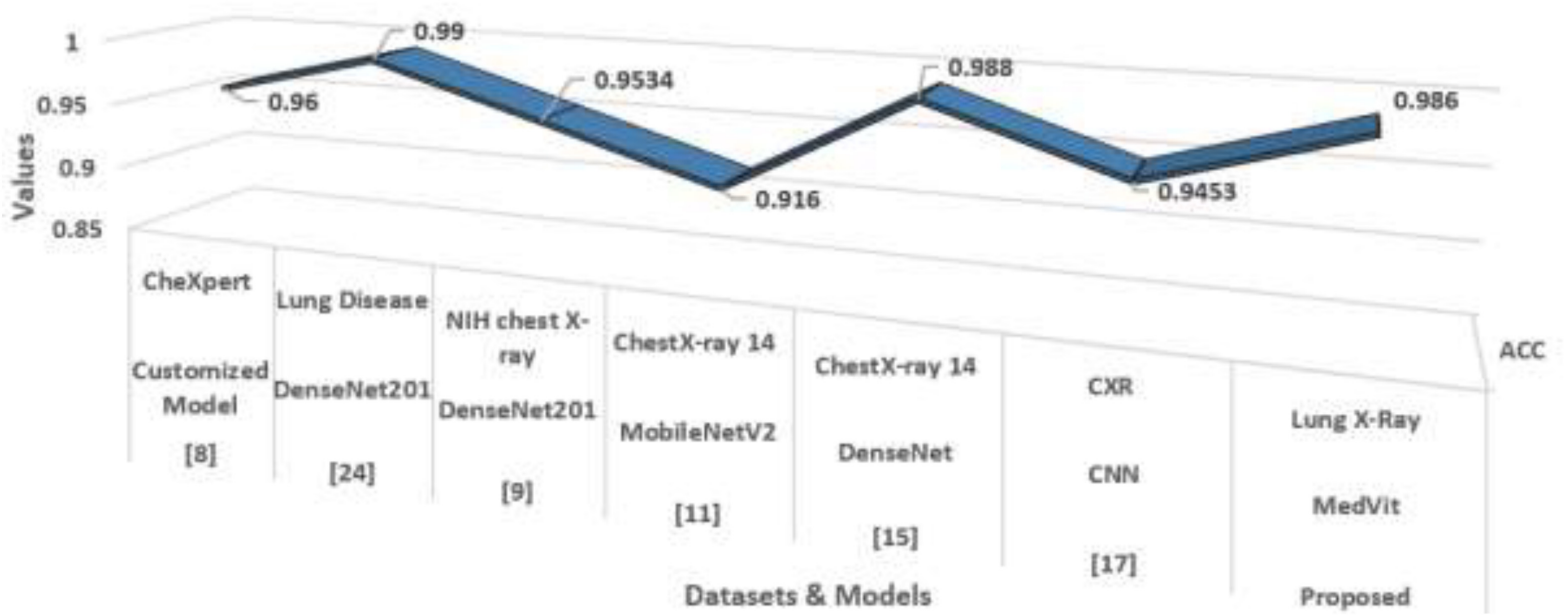
State-of-the-art.

Table 10 and Figure 13 presents a comparative outline of recent state-of-the-art methods for lung infection detection utilizing chest X-ray images. The models assessed span different deep learning structures, including DenseNet, EfficientNet, MobileNetV2, CNN, and GAN-based strategies, over different datasets. Among the reviewed studies, Huy et al. accomplished 98.80 % accuracy with DenseNet on a subset of 5,000 images from the ChestX-ray14 dataset. The proposed MedVit model, connected to a dataset of 10,425 lung X-ray images, illustrated strong performance with an accuracy of 98.6%, putting it competitively among the top-performing models.

**TABLE 10:** State-of-the-art comparison.

References	Technique	Dataset/no. of images	Parameter
Hage Chehade et al. (7)	CycleGAN	ChestX-ray 14/112120	AUC = 91.38%
Patel et al. (8)	Customized efficientnet-B4 and XAI	CheXpert/941	ACC = 96%
Mahamud et al. (24)	DenseNet201	Lung disease/10,000	ACC = 99%
Chutia et al. (9)	DenseNet201	NIH chest X-ray/9,409	ACC = 95.34%
Shamrat et al. (11)	MobileNetV2	ChestX-ray 14/112,120	ACC = 91.6%
Huy and Lin. (15)	DenseNet	ChestX-ray 14/5,000	ACC = 98.80%
Singh et al. (17)	CNN	CXR/5,856	ACC = 94.53%
Proposed	MedVit	Lung X-Ray/10,425	ACC = 98.6%

Patel et al. (8) utilized a customized EfficientNet-B4 design combined with explainable AI (XAI), accomplishing a high accuracy of 96% on the CheXpert dataset, but with a generally small test size of 941 images.

In summary, whereas bigger datasets regularly challenge model execution due to inconsistency and noise, strategies like MedVit have reliably achieved high accuracy. The proposed MedVit model illustrates competitive performance, adjusting accuracy, and versatility, and stands out as an effective approach to more conventional designs in lung infection classification tasks.

## 5 Conclusion

In this study, a novel deep learning-based approach for the classification of lung infections utilizing chest X-ray images is presented, leveraging the capabilities of two advanced transformer-based architectures—MedViT and Swin Transformer. By applying these models to a comprehensive dataset of 10,425 X-ray images categorized into Normal, Lung Opacity, and Viral Pneumonia categories. Furthermore, an in-depth evaluation of different loss functions, namely Hinge Loss, Binary Cross-Entropy, and Kullback-Leibler (KL) Divergence, is conducted to optimize model performance.

- Among the MedViT and Swin Transformer models, the MedViT model appeared to have predominant execution, accomplishing the highest classification accuracy of 98.6% and a minimum loss value of 0.09.
- The Kullback-Leibler (KL) Divergence emerged as the most successful, outperforming both Hinge Loss and Binary Cross-Entropy with an achieved value of accuracy of 98.5%.

This study illustrates that combining transformer-based models with a focus on information security and cybersecurity considerations can improve the reliability and clinical appropriateness of automated lung illness determination frameworks. This research lays the foundation for future improvements in AI-assisted medical imaging and underscores the practical relevance of receiving hybrid deep learning models to support clinical decision-making with more prominent accuracy and reliability. Future work can focus on expanding disease classes, incorporating explainable AI, 3D imaging, and multi-modal analysis. In future research, integration of federated learning frameworks is planned to enable privacy-preserving training across distributed clinical datasets and enhance scalability. Also, future work will investigate the execution of blockchain-based systems to supply tamper-proof logging and secure sharing of medical imaging information, and advance reinforcing the cybersecurity framework of AI-assisted diagnostic systems. In future work, a collaboration with medical institutions can be done to test the model on real hospital-acquired images and assess its robustness across diverse patient populations and imaging conditions.

## Data Availability

The original contributions presented in the study are included in this article/supplementary material, further inquiries can be directed to this corresponding author.
